# Dysregulated Striatal Neuronal Processing and Impaired Motor Behavior in Mice Lacking Huntingtin Interacting Protein 14 (HIP14)

**DOI:** 10.1371/journal.pone.0084537

**Published:** 2013-12-23

**Authors:** Ana María Estrada-Sánchez, Scott J. Barton, Courtney L. Burroughs, Amanda R. Doyle, George V. Rebec

**Affiliations:** Program in Neuroscience and Department of Psychological and Brain Sciences, Indiana University, Bloomington, Indiana, United States of America; University of Florida, United States of America

## Abstract

Palmitoyl acyl transferases (PATs) play a critical role in protein trafficking and function. Huntingtin interacting protein 14 (HIP14) is a PAT that acts on proteins associated with neuronal transmission, suggesting that deficient protein palmitoylation by HIP14, which occurs in the YAC128 model of Huntington’s disease (HD), might have deleterious effects on neurobehavioral processing. HIP14 knockout mice show biochemical and neuropathological changes in the striatum, a forebrain region affected by HD that guides behavioral choice and motor flexibility. Thus, we evaluated the performance of these mice in two tests of motor ability: nest-building and plus maze turning behavior. Relative to wild-type controls, HIP14 knockout mice show impaired nest building and decreased turning in the plus maze. When we recorded the activity of striatal neurons during plus-maze performance, we found faster firing rates and dysregulated spike bursting in HIP14 knockouts compared to wild-type. There was also less correlated firing between simultaneously recorded neuronal pairs in the HIP14 knockouts. Overall, our results indicate that HIP14 is critically involved in behavioral modulation of striatal processing. In the absence of HIP14, striatal neurons become dysfunctional, leading to impaired motor behavior.

## Introduction

Palmitoyl acyl transferases (PATs) bind palmitic acid, a 16-carbon lipid, to specific cysteine residues, a post-translational modification known as palmitoylation [1]. Protein palmitoylation increases hydrophobicity to facilitate protein insertion into membranes and plays a role in protein-protein interactions and the clustering and trafficking of proteins [1,2]. In the brain, the mobilization and operation of membrane proteins such as neurotransmitter receptors and transporters depends on protein palmitoylation [3-5]. Thus, disruption of this mechanism can have detrimental effects on synaptic function and neuronal plasticity [5]. Previous studies have associated impaired palmitoylation with schizophrenia, X-linked mental retardation, and Huntington’s disease (HD) [4,6-10]. Despite mounting evidence for the importance of palmitoylation in brain function, its role in behavior and behavior-related neuronal processing has not been evaluated. 

Huntingtin interacting protein 14 (HIP14) palmitoylates key synaptic proteins such as glutamic acid decarboxylase (GAD) GAD-65, the synthesizing enzyme for gamma-aminobutyric acid (GABA), an inhibitory neurotransmitter, and postsynaptic density protein (PSD) PSD-95, a scaffold protein associated with receptor clustering and signaling during synaptic transmission [11-14]. Palmitoylation is impaired in HIP14 knockout mice, which show neuropathological changes similar to those described for transgenic models of HD, including decreased striatal volume and atrophy of medium spiny neurons (MSNs), the most abundant neuronal type in the striatum [15]. HD, an autosomal dominant condition caused by a mutation in the huntingtin (HTT) protein, is characterized by a progressive loss of motor, cognitive, and emotional control [16,17]. Interestingly, HD transgenic models develop behavioral, neuropathological, and biochemical changes resembling those described in HD patients. Electrophysiological studies of behaving transgenic mouse models of HD indicate that long before the phenotype emerges, patterns of neuronal communication become dysfunctional [18-20]. Because mutant HTT impairs HIP14 function, which prevents the proper HIP14 palmitoylation of several synaptic proteins [4,10,15], altered electrophysiological properties of presumed MSNs may be due in part to a failure of HIP14 activity. Here, we evaluated the firing patterns of individual striatal neurons in HIP14 knockout and wild-type mice while they explored a plus maze, a behavioral test that provides information about arm choice patterns to identify motor deficits such as behavioral inflexibility [21]. We also evaluated nest-building performance, a behavior related to thermoregulation and shelter that involves motor coordination and depends on striatal function [22,23]. 

## Materials and Methods

### Animal housing and genotype

We attest that all efforts were made to minimize the number of animals used and their suffering, and certify that animal use was approved by the Institutional Animal Care and Use Committee of Indiana University based on guidelines established by the National Institutes of Health. HIP14 knockout and wild-type littermates (FVB/N background strain) were bred from heterozygous pairs obtained from the Hayden laboratory at University of British Columbia, Vancouver, Canada. Mice were housed individually and maintained under controlled temperature and humidity conditions with a 12-h light/dark cycle and food and water ad libitum. Genotyping was carried out on DNA extracted from tail tissue placed in 25 μL cell lysis buffer (50 mM Tris, pH 8.0; 25 mM EDTA; 100 mM NaCl; 0.5% IGEPAL CA-630; 0.5% Tween 20) and proteinase K (10 mg/mL; 60 μg/reaction) and incubated at 55°C overnight. DNA was diluted with 350 μL filter-sterilized water, heated to 100°C for 10 min, centrifuged for 2 min at 17,000 X *g*, and stored at 4°C. Polymerase chain reaction (PCR) and agarose gel electrophoresis were used to determine genotype. The following primers were used: Hip 14Int5F (5’- CCG TCT TAG TGC CAT TTG TTC GTC-3’); BetaGeo5’R (5’-GGT GCC GGA AAC CAG GCA AAG-3’); and Hip14Int5A (5’-CAT GTG TCG GGA TGG CTG TGA AAA G-3’). Each reaction consisted of 2.0 μL DNA template, 0.4 μL each primer (20 μM stock solution), 7.2-μL filter-sterilized HPLC water, and 10.0 μL 2x Biomix Red (Bioline USA, Taunton, MA) for 20 μL total volume. For PCR cycling, samples were maintained at 94°C for 3 min followed by 30 cycles at 94°C for 30 s, 62°C for 45 s, and 72°C for 60 s, with a final elongation at 72°C for 7 min. Electrophoresis was performed in type I agarose with 0.5 μg/mL ethidium bromide at 5 V/cm for 180 min using a 100-base pair ladder as DNA standard. Gels were evaluated with Kodak Image Station 4000R and Kodak Molecular Imaging software (Carestream Molecular Imaging, New Haven, CT) to confirm genotype. A single 600 bp band indicated the wild-type condition, and a single 480 bp band indicated the homozygous HIP14 knockout genotype. 

### Nest-building behavior

 Nest building behavior was assessed according to the protocol described by Deacon (2006) [24]. Once weekly, a new 5 cm pressed cotton square Nestlet® (Ancare, Bellmore, NY, USA) was weighed and placed in the home cage of each mouse. On each of the three days following the placement of the Nestlet®, nesting quality was assessed on a scale of 1-5: 1 = material was mostly unused, with >90% intact; 2 = material was partially used, with 50% to 90% intact; 3= the material was torn apart and scattered with <50% intact, but there was no detectable nest site; 4 = most (>90%) of the material was used to make a discernible but flat nest; and 5 = most (>90%) of the material was used to build a complete nest having a central cavity and walls higher than the body height of the mouse. The mass of the remaining unused Nestlet® was also weighed on each of the three consecutive days after its placement, and the percentage of used material was then calculated. HIP14 knockout and wild-type mice nest building behavior was monitored from 12 to 36 weeks of age. Some mice used for the nest building study were subjected to electrode surgery implantation (described below) to evaluate the electrophysiology properties of striatal neurons. 

### Electrode implantation surgery

Each electrode bundle consisted of four 50 μm Formvar-insulated stainless steel wires (California Fine Wire, Grover Beach, CA) and one 50 μm uninsulated stainless steel ground wire. Two bundles were friction-fitted to gold pin connectors in a custom nylon hub (6-mm diameter). The electrode assembly is small, lightweight, and well tolerated by the mice so that they could behave freely.

Mice were weighed prior to surgery implantation. At 32 weeks of age, HIP14 knockout mice showed a significant reduction in body weight (26.7±1.0 g, N=12) compared to wild-type littermates (32.8 ± 1.3g, N=14; t=3.3, df=24, p = 0.002). Mice were anesthetized with an IP injection of a mixture of chloral hydrate and sodium pentobarbital or chloropent (170mg/kg chloral hydrate, 40mg/kg sodium pentobarbital; 0.4ml/100g body weight) and mounted in a stereotaxic frame. Following a midline scalp incision, a hole was drilled through the skull to target the striatum (+ 0.8 mm anterior and ± 2.2 mm lateral to bregma and 3.2 mm ventral) [25]. Two additional holes were drilled in the contralateral site for stainless anchor screws. Electrodes were lowered into the striatum and the electrode assembly was then permanently attached to the skull by means of dental acrylic. Mice were allowed 1 week of postsurgical recovery in individual cages with food and water ad libitum.

### Plus maze

Behavioral performance in the plus maze was evaluated simultaneously with electrophysiological activity for both HIP14 knockout and wild-type mice. The plus maze was suspended 2 mm above a force-plate actometer, a device that monitors the position of the mouse and indicates the number of turns into each arm (right, left, front and back) [26]. Plus-maze turning behavior is a measure of motor flexibility, or the probability of turning, determined by the sum of arm choices to the right and left arm divided by the total number of choices (left, right, and front) [21,26]. Once a mouse was connected to our recording system, it was placed in one arm of the plus maze and allowed to explore the maze freely for 30 min. Typically, mice explore the first arm until they reach the center or choice point, where they have the option to continue straight to explore the opposite arm or turn to enter either the right or left arm [21,26]. 

### Electrophysiology

Neuronal activity was recorded during the light phase of the diurnal cycle while the mice freely explored the arms of a plus maze as described elsewhere [21]. Recording sessions were conducted once weekly for 30 min each when the mice were between 32 and 52 weeks of age. The electrode assembly was connected to a lightweight flexible wire harness equipped with field-effect transistors that provided unity-gain current amplification for each of the eight microwires. Neuronal discharges were acquired by Multichannel Acquisition Processor (MAP) through a preamplifier (Plexon, Dallas, Texas). The MAP system allows for direct computer control of signal amplification, frequency filtering, discrimination, and storage. To detect spiking activity, signals were band-pass filtered (154 Hz to 8.8 KHz) and digitized at a rate of 40 KHz. All spike sorting occurred online before the beginning of the recording session. Sort Client software (Plexon, Dallas, Texas) was used in conjunction with oscilloscope tracking to isolate each unit (matching the analog signal with the digitized template) and to eliminate the need for post hoc off-line sorting. Voltage threshold 2.5-fold background noise was established and a template waveform was created via component analysis. Autocorrelation and interspike interval (ISI) analyses for each unit were used to avoid recording the same unit on multiple channels. Mice participated in multiple recording sessions. Recorded units were treated as independent entities in each recording session because electrode drift and subtle changes in behavioral state cannot guarantee positive detection of the same neuron over multiple sessions [27]. 

### Spike train analysis

Electrophysiological data for each recording session was analyzed by means of NeuroExplorer (Nex Technologies, Littleton, MA). Analysis was performed on single-unit data collected over the entire 30-minute recording session. Firing rate was calculated by dividing the spike trains into 1-s bins (spikes/s). To assess spike-train variability, the coefficient of variation of interspike intervals (CV ISIs) was calculated by dividing the standard deviation of all ISIs in a train by the mean ISI of the train. Bursting activity, which corresponds to periods of high-frequency firing, was calculated by the Poisson surprise method [18,28]. This method uses a probability-based approach to burst detection that compares successive ISIs in a spike train to a Poisson spike train with the same firing rate. If a set of consecutive ISIs occurs with a sufficiently low probability, the event is considered “surprising” and classified as a burst. Therefore, the surprise value is an index of how intense or “surprising” the ISIs of a particular burst are compared with other ISIs in the same train, and provides an estimate of the statistical significance of each burst in the train [18]. Burst surprise is not sensitive to fluctuations in average firing rate and treats each spike as an independent entity. Moreover, this method is well established for detecting burst activity in striatum [29-31]. This analysis requires setting a minimum surprise value, and here we used a value of 5, which estimates that a burst occurs about 150 times (P=0.007) more frequently than would be expected in a Poisson spike train with the same mean firing rate [32]. The following bursting properties were analyzed for each recorded neuron: burst rate, percent of all recorded spikes that occurred in bursts, mean burst surprise, mean burst duration, mean ISI in a burst, mean burst frequency, and mean number of spikes per burst. 

We also compared one-second intervals of neuronal activity at the choice point with activity obtained at any arm of the plus maze. For this purpose, a total of 50 random events were obtained one second before the mice entered the choice point. For comparison, 50 random events (also one-second duration) were taken two seconds after the mouse left the center of the maze. These events were obtained through NeuroExplorer. 

To determine the percentage of coincident bursts, defined as bursts from two simultaneously recorded neurons [33], the number of burst overlaps were divided by the sum of bursts between the two units. The mean time (duration) that bursts were coincident was also calculated. Coincident bursting and coincidence duration were determined for each pairwise comparison in each session. To assess correlated activity between two spike trains, cross-correlation histograms (CCHs) were constructed for each pairwise comparison [34] for each 30-minute recording session. All CCHs were based on 0.5 ms bins and a 0.5-s time lag from the zero bin. CCHs were smoothed using a Gaussian filter with a bin width of 3. Significant peaks, indicating correlated firing in both the raw and smoothed CCHs, were indentified using a 99% confidence interval by assuming the null hypothesis that each spike train is a Poisson process and that firing between neuronal pairs is independent [35]. 

### Histology

Electrode placement was verified after the final recording session was completed in each mouse by deeply anesthetizing the mouse with chloropent and a running current pulse (30 μA for 10 s) through each active microwire of each electrode bundle. Mice were perfused and brains preserved as previously described [18]. Consecutive series of striatal coronal sections of 40 μm were obtained by cryostat and mounted in gelatin-subbed slides. The sections were stained with cresyl violet and examined under a light microscope to confirm electrode bundle location, which was identified as a clear blue spot; only recordings that had electrode placements verified in the striatum were included in the analysis. 

### Statistical analysis

Statistic program package GraphPad Prism 6 (GraphPad Software, San Diego, CA) was used for statistical analysis of behavioral and electrophysiological data. Corporal body weight was analyzed by an unpaired t test, which was also used to analyze plus-maze turning. Nest-building behavior was analyzed by two-way repeated measures ANOVA (RM ANOVA) followed by Bonferroniʼs multiple comparisons test. Nonparametric statistics were used to analyze the electrophysiology data because of significant deviation from normality and a lack of homogeneous variances in spike-train samples [36]. Thus, we used the Mann-Whitney U test to compare firing rate, CV ISI, and all bursting parameters. Our electrophysiology data are presented as box-and-whiskers plots; the box extends from the 25th to the 75th percentile with the line at the median (50^th^ percentile), and the whiskers represent the minimum and maximum values. A chi-square test with Yates correction was used to determine differences in the proportions of correlated and non correlated neurons. Two-way ANOVA was used to compare neuronal activity observed when the mice were at the choice point versus in the plus-maze arm. Data is expressed as mean ± SEM; differences were considered significant when p ≤ 0.05.

## Results

### Behavior

#### Nest-building activity

**Figure 1:**
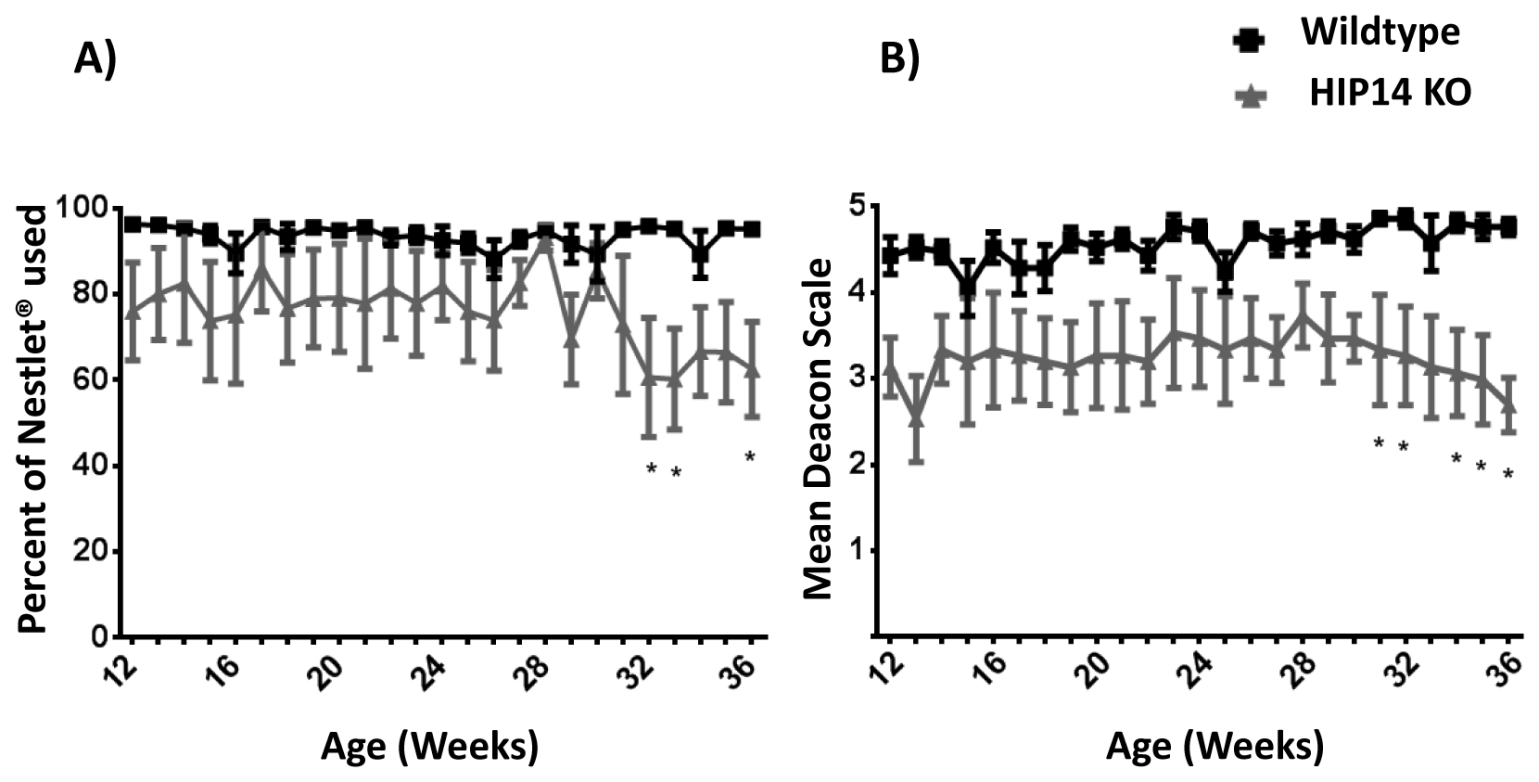
Impaired nest building performance in HIP14 knockout mice. Nest building activity was evaluated from 12 to 36 weeks of age. Two characteristics were studied: (A) the percentage of Nestlet® used and (B) the completion of the nest according to Deacon score (see material for score description). Data were analyzed through repeated measure two-way ANOVA and Bonferroniʼs multiple comparisons test. N=7 and N=5 for wild-type and HIP14 knockout, respectively. DATA represented as MEAN ± SEM. * p=0.04.

 Nest-building behavior was studied in wild-type and HIP14 knockout mice from 12 to 36 weeks of age (N=7 wild-type and N=5 HIP14 knockout). Two characteristics of nest building were evaluated: the amount of Nestlet® used and the level of completion of the nest. Figure 1A shows that wild-type mice mostly used the entire Nestlet® throughout the study. In contrast, HIP14 knockout mice tended to use less Nestlet® material. A two-way repeated measures ANOVA revealed a significant difference between genotypes (F_(1, 10)_=8.66; p=0.014). However, no interaction between genotype and age were observed (F _(24, 240)_=1.39, p=0.10). Although HIP14 knockouts tended to use less Nestlet® material at early ages, a significant difference relative to wild-type is observed from 32 weeks of age (Figure 1A: t= 3.51; p=0.012). Figure 1B shows nest construction. Whereas wild-type mice build a relatively complete nest at all ages studied, HIP14 knockout mice consistently build a poor quality nest: flat without a discernible nest site. A two-way repeated measures ANOVA revealed a significant difference on nest construction between genotypes (F_(1,10)_=11.7, p=0.006). Note that the HIP14 knockout nest is deficient as early as12 weeks of age, and statistical differences in nest construction relative to wild-type is evident through 31 weeks of age (t=3.13; p=0.04).

#### Plus maze

 Wild-type mice often turn to explore the perpendicular arms (probability of turning is 0.71±0.01; N=38). In contrast, HIP14 knockout mice are less likely to turn (probability of turning is 0.60±0.02, N=46); instead, once they cross the choice point, they explore the opposite arm, a common sign of motor inflexibility [21]. Statistical analysis of probability of turning reveals a significant difference between wild-type and HIP14 knockout mice (t=3.6, df=82, p=0.0005).

### Electrophysiology

#### Striatal firing pattern

**Figure 2:**
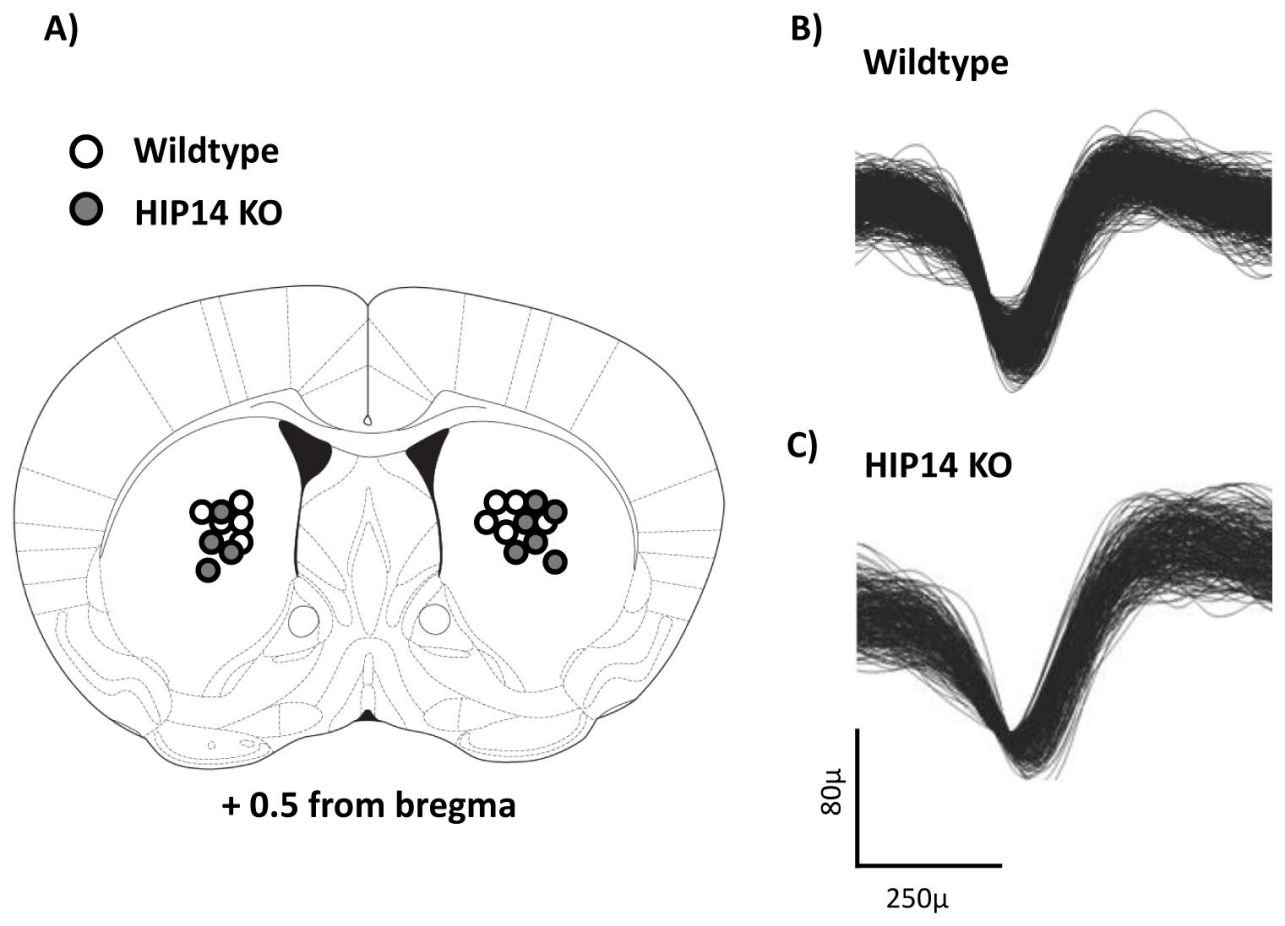
Electrode position and medium spiny neuron (MSN) waveforms in wild-type and HIP14 knockout mice. A) Electrode position was verified through histological analysis, the coronal section of mouse brain showing the position of each electrode bundle in wild-type (white) and HIP14 knockout (gray) mice. Sample of waveforms collected from a putative MSN from the striatum of a wild-type (B) and HIP14 knockout (C) mouse.

Histological analysis confirmed that all microelectrode placements were in the striatum of both groups of mice, as shown schematically in Figure 2A. Spike waveforms of wild-type and HIP14 knockout mice are shown in Figures 2B and C, respectively. All recorded units displayed waveforms and firing patterns characteristic of MSNs recorded in vivo, as previously described [18,20]. 

**Figure 3:**
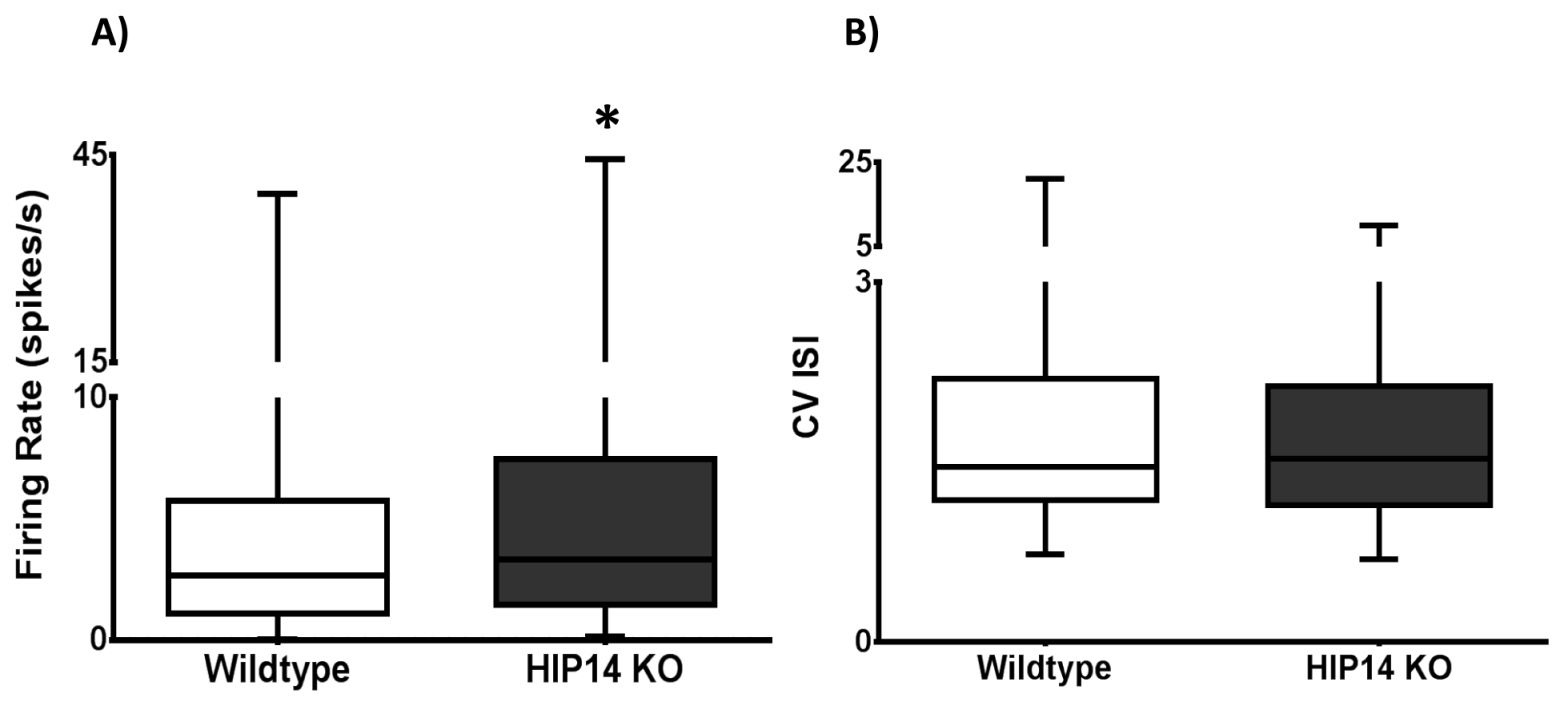
Increased spike activity in the striatum of wild-type and HIP14 knockout mice. Neuronal activity was collected while mice freely explored the plus maze for 30 min. Data are illustrated as box-and-whiskers plots. The box extends from the 25th to the 75th percentile with the line indicating the median (50^th^ percentile). The whiskers represent minimum and maximum values. A) Firing rate is expressed as the number of spikes per second; B) Coefficient of variation of inter-spike intervals (ISIs). N=131 neurons in wild-type and N=146 in HIP14 knockout. Data were analyzed by Mann-Whitney U test. p=0.032 for firing rate and p=0.77 for CV ISI.

Striatal neuronal activity was recorded while mice explored the plus-shaped maze for 30 minutes. We recorded a total of 131 units in 14 wild-type and 146 units in 12 HIP14 knockout mice. Figure 3A shows that the firing rate (spikes/s) of HIP14 knockout neurons is higher than that of wild-type neurons (p=0.032), although evaluation of the CV ISI indicated no group difference in the variability of the spike train (Figure 3B; p=0.77). 

**Figure 4:**
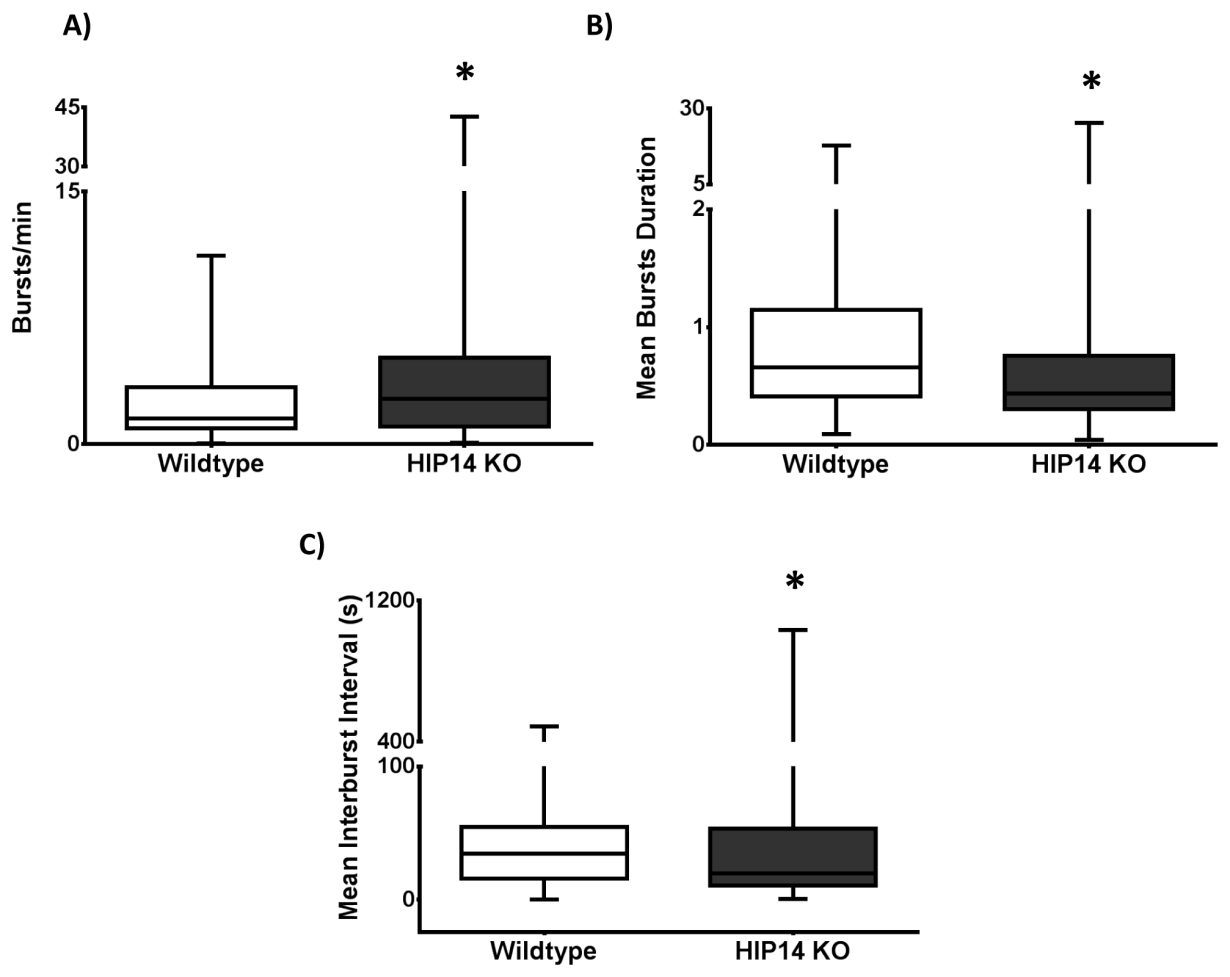
Striatal neurons in HIP14 knockouts show impaired bursting activity. A) Bursting activity as expressed by number of bursts per minute B) mean burst duration C) Mean interburst interval. Data were analyzed by Mann-Whitney U test. p<0.005 .

**Figure 5:**
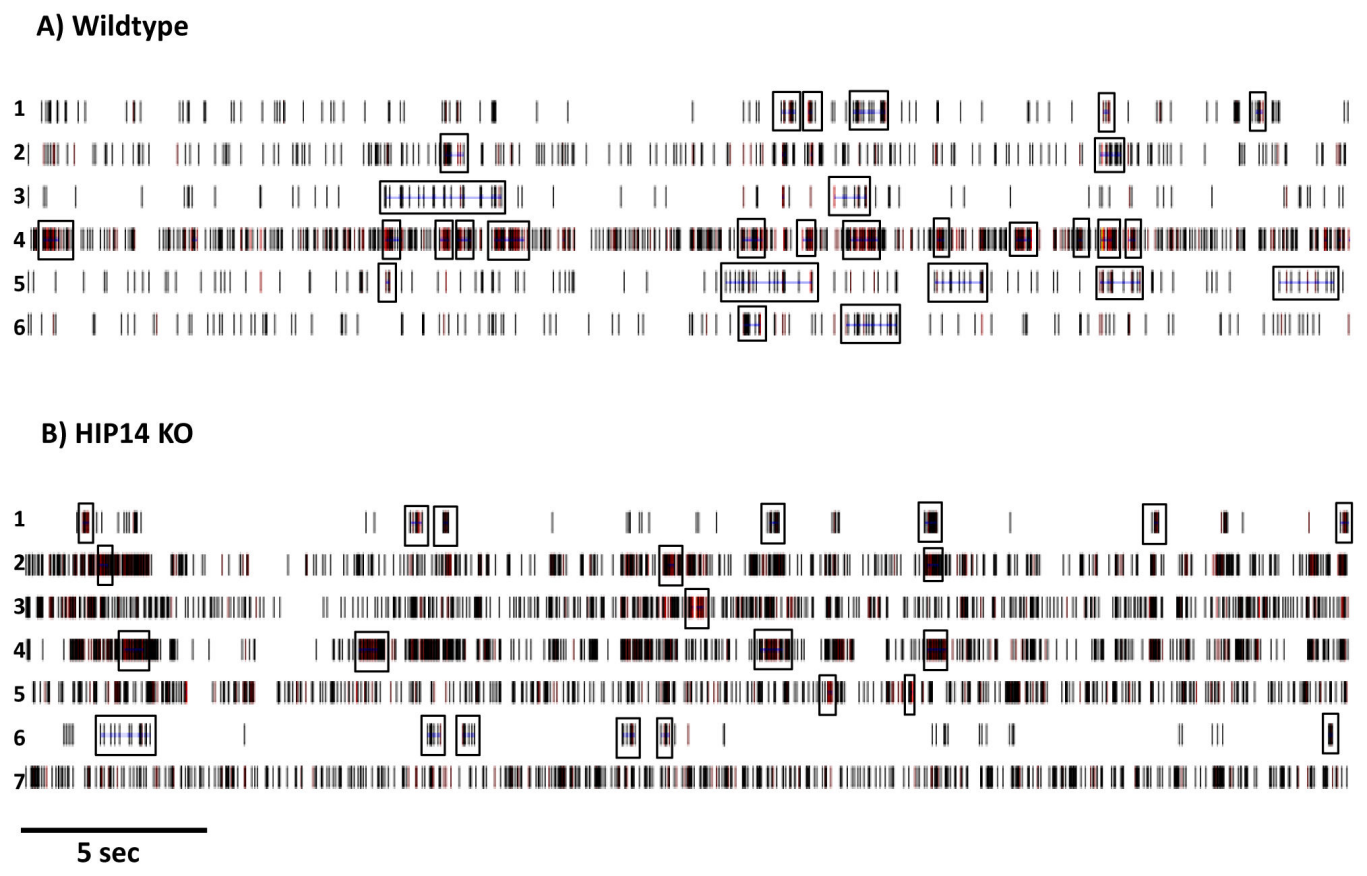
Sample spike train rasters (30 s) from wild-type and HIP14 knockout striatal neurons. Example of six neurons simultaneously recorded in the striatum of wild-type (A) and seven units recorded in HIP14 knockout mice (B). Bursts are denoted by boxes.

 Burst firing is disrupted in the HIP14 knockouts. In these mice, bursts occur more often than in wild-type mice (Figure 4A; p=0.005) and also have a shorter duration (p=0.0001) and a reduced interval between bursts (p=0.003; Figure 4B and C, respectively). Other burst properties, such as the percentage of spikes that participate in a burst and the burst surprise value, did not differ from wild-type (data not shown). Differences in firing rate and bursting can be observed in the spike train rasters sampled from wild-type and HIP14 knockout mice in Figure 5. Note the increased firing rate, the increased number of bursts, and the short duration of each burst in HIP14 knockout versus wild-type units. 

Neuronal activity was also evaluated at two specific points: a) one second before the mouse enters the choice point and b) two seconds after it leaves the choice point and is exploring any arm of the maze. Firing patterns are summarized in Table 1. Both wild-type and HIP14 knockout striatal neurons show similar number of spikes and firing rate when mice are in either the choice point or the arm of the maze. Relative to neuronal activity observed at the choice point, striatal neurons in wild-type mice display increased number of bursts, mean burst duration, and mean interburst interval when they explore the arm. Similar changes in burst firing are observed in HIP14 knockout striatal neurons (see Table 1). 

**Table 1:** Striatal Neuronal activity in wild-type and HIP14 knockout mice at the choice point and arm.

			**Wildtype**		**HIP14 KO**	
Electrophysiological parameters			Choice Point	Arm	Choice Point	Arm
Spikes			197.7±29.18 †	225.1±29.7 †	311.0±33.6	323.3±32.4
Mean frequency			4.5±0.6 **†**	4.8±0.5	6.3±0.6	6.5±0.6
# Burst			1.2±0.1* **†**	1.9±0.2 **†**	2.1±0.2 *	2.8±0.2
% Spikes in Burs			9.5±1.4 **†**	14.0±1.7 **†**	14.2±1.7 *	19.7±1.9
Mean Burst Duration			0.11±0.01*	0.16±0.01	0.13±0.01 *	0.20±0.01
Mean Spikes in Burst			6.2±1.2	7.7±1.1	*7.8±0.9*	10.1±0.9
Mean Interburst Interval			104.2±23.8*	205.8±30.7	*160.7±24.3*	218.3±28.6
Mean Burst Surpise			5.2±0.9	5.6±0.7	*5.9±0.7*	7.2±0.7

^*^ Statistically different relative to neuronal activity obtained while mice explore the arm p< 0.05.

^†^ relative to neuronal activity observed in HIP14 knockout p< 0.05.

Data expressed as MEAN±SEM. N=126 events in wild-type and N=133 in HIP14 knockout mice.

Interestingly, important differences occur when comparing neuronal activity between wild-type and HIP14 knockout mice when they explore either the center or the arm of the maze. At the choice point, HIP14 knockout neurons show increased firing rate, increased number of bursts, and a greater number of spikes per burst relative to the wild-type group. When HIP14 knockout mice explore the arm, there are prominent changes in bursting properties, but no difference in firing rate frequency (Table 1). 

#### Correlated firing

**Figure 6:**
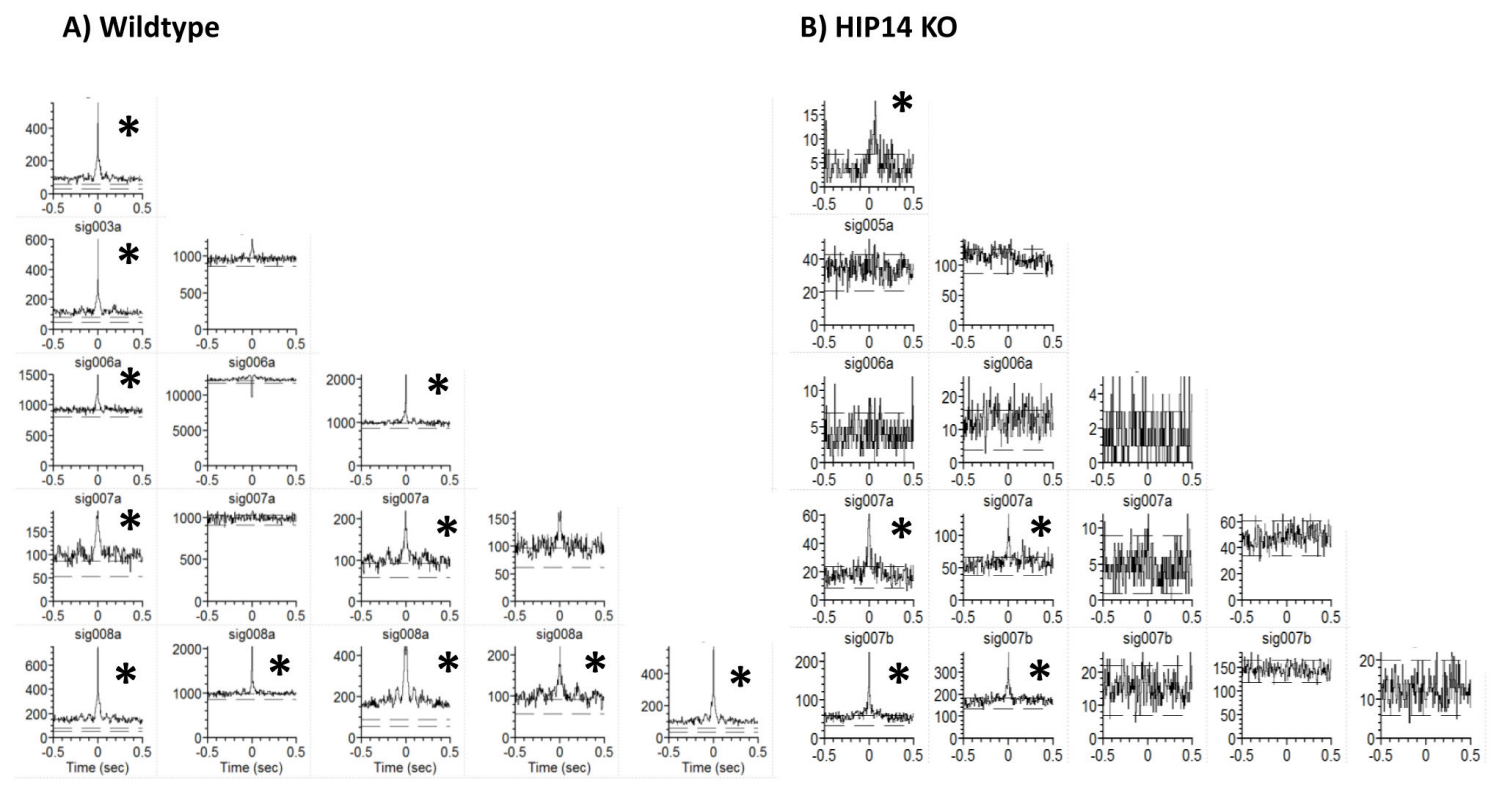
Representative cross-correlation histograms in wild-type (A) and HIP14 knockout mice (B). CCHs were generated by five units each from wild-type and HIP14 knockout mice. Each correlation matrix shows all possible pairs (bin width =0.05 s). Correlated neurons can be recognized by the presence of peaks that exceed the 99% confidence limit. Note the reduced number of correlated unit pairs in the HIP14 knockout matrix; each correlated pair is denoted by asterisk.

**Figure 7:**
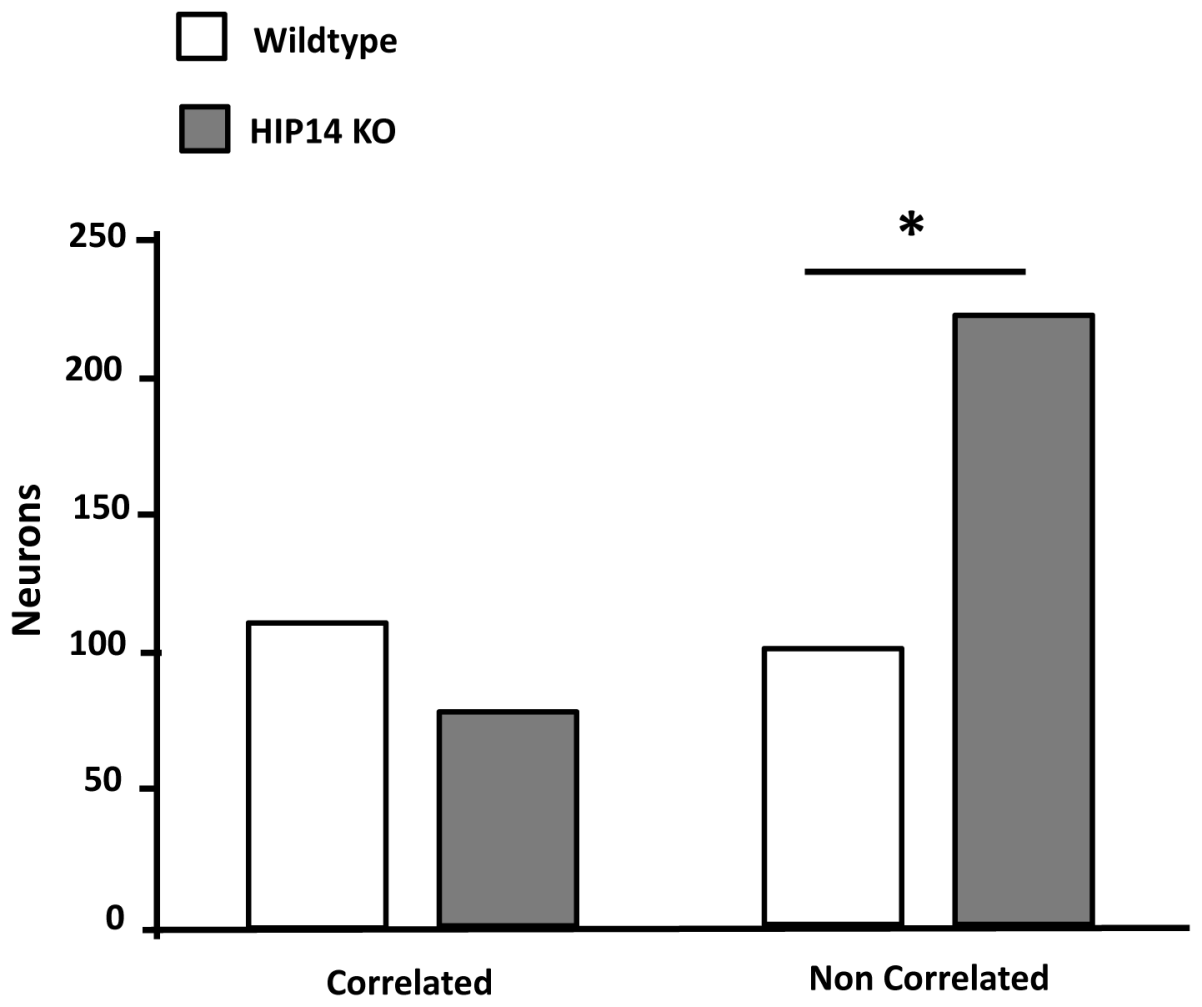
Reduced correlated neuronal activity in the striatum of HIP14 knockout mice. The graph shows the total number of correlated and non-correlated neurons in the striatum of wild-type and HIP14 knockout mice. Data were analyzed by Chi-square test with Yates correction. χ^2^=36.7, df=1, p=0.0001.

The proportion of correlated pairs of simultaneously recorded neurons was evaluated for each recording session, as previously described [18]. Correlated firing between pairs of units occurs when the CCH confidence limit exceeds 99% [35]. Sample CCHs for wild-type and HIP14 knockout mice are shown in Figure 6. Note the large proportion of unit pairs that showed correlated firing (denoted by asterisk) in wild-type striatum (Figure 6A) compared with the HIP14 knockout (Figure 6B). From a total of 187 neuronal pairs recorded in wild-type mice, 110 were correlated (58.8 % of total pairwise of neurons) and 77 (41.1%) were not. In the HIP14 knockout group, 320 total pairs were recorded, and only 99 (30.9%) showed correlated activity while 221 (69%) did not. Our χ^2^ analysis indicated that these group differences were highly significant (Figure 7; χ^2^ =36.7; df=1, p=0.0001), suggesting that the HIP14 knockout disrupts behavior-related correlated firing in striatum.

**Figure 8:**
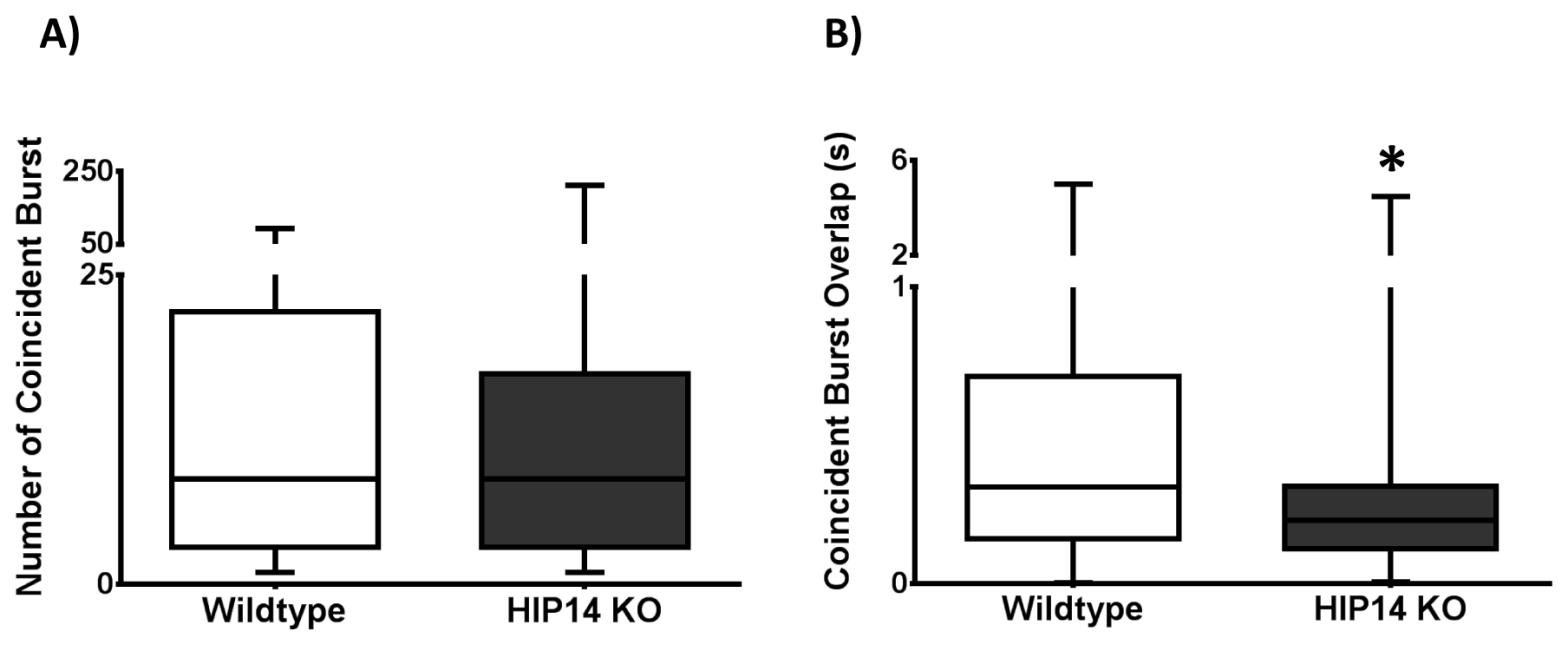
Bursting activity in pairwise neurons in the striatum of wild-type and HIP14 knockout mice. A) Coincident burst. B) Coincident burst overlap. Data were analyzed by Mann-Whitney U test. p<0.0001.

We also assessed coincident bursting between neuronal pairs, and although there was no difference in the number of coincident bursts between HIP14 knockout and wild-type mice (Figure 8A), we found a significant decrease in the amount of overlap between bursting pairs in HIP14 knockout mice (P<0.0001; Figure 8B), consistent with a decrease in burst duration.

## Discussion

We demonstrate here that HIP14 knockout mice show deficient nest-building behavior and reduced plus-maze turning relative to wild-type littermates. Moreover, striatal neuronal processing is impaired in HIP14 knockouts as manifested by increased firing rate and altered bursting. These mice also show a decrease in correlated firing between simultaneously recorded neuronal pairs. Together, these results indicate that protein palmitoylation by HIP14 is a key component for behavior-related neuronal processing in the striatum. 

For nest building, healthy mice began by shredding the Nestlet® material, relying on the coordinated activity of forelimbs and orofacial muscles [23]. HIP14 knockouts leave increasing amounts of the material unused as they age, a sign of impaired motor coordination. In fact, tests aimed at motor coordination (e.g., fixed and accelerating rotarod) show deficits in HIP14 knockouts [15]. Such deficits also may interfere with actual construction of the nest, as demonstrated in HIP14 versus wild-type mice. However, poor nest construction could also reflect deficiencies in synaptic plasticity related to cognitive processes. This hypothesis is consistent with evidence that HIP14 palmitoylates PSD-95, a scaffolding protein associated with the recruitment of N-methyl-D-aspartate (NMDA) receptors required for spatial memory acquisition [37,38]. In fact, reduced palmitoylation of PSD-95 occurs in the brain of HIP14 knockouts [39]. Thus, it is possible that impaired synaptic plasticity in the striatum might underlie poor nest construction observed in HIP14 knockout mice. 

A change in the dopaminergic system also may contribute to impaired nest building in HIP14 knockout mice. Szczpka et al. (2001) reported that dopamine-deficient mice are not able to construct a nest, and restoration of dopamine synthesis in the striatum re-established nest-building behavior [22]. Similarly, this behavior is impaired by systemic injection of the dopaminergic toxin 1-methyl-4-phenyl-1,2,3,6-tetrahydropyridine (MPTP) [40]. Previous research indicated that HIP14 knockout mice display significant but non-progressive striatal volume loss and reduced neuronal counts, particularly affecting the MSNs expressing enkephalin and dopamine- and cyclic AMP-regulated phosphoprotein (DARPP32) neurons [15]. Several dopaminergic proteins, such as the D1 and D2_L_ dopamine receptors and the dopamine transporter are targets of protein palmitoylation [41-44], and although the PAT responsible for their palmitoylation has not been identified, it seems likely that dysfunctional dopaminergic neurotransmission could play a role in impaired nest building in HIP14 knockouts. 

The low probability of turning into the left or right arm as mice explore the plus maze has been linked to motor inflexibility, which is often observed in HD transgenic models [21]. Along with impaired motor behavior, we identified abnormal patterns of striatal activity in HIP14 knockout mice at the choice point, where the mouse has to decide if it will continue straight to explore the opposite arm or turn to either the right or left arm (see Table 1). At the choice point, HIP14 knockout striatal neurons show increased firing rate and bursting activity, which may impact specific behaviors such as turning. Therefore, aberrant striatal processing observed at the choice point might relate to the low probability of turning in HIP14 knockout mice, due to changes in firing rate and bursting activity that might modify neuronal transmission related to reliability of information transmission [33,45]. Moreover, simultaneously recorded neurons in HIP14 knockout mice tend to operate in a non-correlated way, which may also play a role in motor inflexibility because synchronous neuronal activity shapes behavioral output [46-48]. This information suggests that protein palmitoylation by HIP14 plays a critical role in striatal processing and appears necessary for normal motor output.

A significant decrease in the number of excitatory synapses has been reported for the striatum of HIP14 knockout mice, similar to what has been described for the full-length HD transgenic model YAC128 [15]. These data suggest that impaired cortical input, which has been implicated in striatal dysfunction in HD models [49], may contribute to dysregulated MSN processing in HIP14 knockout mice. Moreover, MSNs release GABA and give rise to the direct and indirect striatal output systems. The direct pathway projects to the internal segment of globus pallidus (GPi) and the substantia nigra pars reticulata (SNr), both of which comprise the output structures of the basal ganglia and project to brainstem and thalamus. The indirect system also targets the GPi and SNr but through synaptic connections in the external segment of the globus pallidus (GPe) and the subthalamic nucleus (STN). The striatum is the first information processing unit of the basal ganglia, and together with the thalamus and cerebral cortex, constitutes the neuronal circuitry that shapes motor control. It is possible that altered MSN activity in HIP14 knockout mice leads to altered brain activity downstream from the striatum, which might underlie dysfunctional motor behavior and other striatum-related behaviors. This is a likely possibility because HIP14 palmitoylates GAD 65, the enzyme that catalyzes the synthesis of GABA, the neurotransmitter released by MSNs to downstream targets [12,13]. Moreover, dysfunctional neurotransmitter release might also contribute to deficient neurotransmission in HIP14 knockouts, since HIP14 palmitoylates SNAP-25 and synaptotagmin I, two proteins involved in the fusion of presynaptic vesicles containing the neurotransmitter [12]. Similar findings have been reported for other HIP14 knockout organisms. For example, recordings of excitatory junctional potentials in the Drosophila melanogaster HIP14 mutant show impaired evoked neurotransmitter release [50,51]. 

Recent studies indicate that in addition to its PAT activity, HIP14 mediates Mg^2+^ transport [52,53] and although the impact of this mechanism on neuronal transmission has yet to be evaluated, a change in Mg^2+^ homeostasis may impact striatal processing. Consider that MSNs receive inputs from cerebral cortex that release glutamate. The Mg^2+^ that blocks the glutamatergic receptor NMDA is released upon membrane depolarization, allowing full NMDA receptor activation. Therefore, changes in Mg^2+^ concentration might affect NMDA function. Further studies need to be done to understand the role of HIP14 as a Mg^2+^ transporter and its relation with neuronal transmission. 

Proper HIP14 activity depends on its interaction with the HTT protein [54]. Here we show that HIP14 knockouts show signs of motor inflexibility that often occur in HD transgenic models [21]. At the electrophysiological level, HD transgenic mice show impaired striatal neuronal processing [18,19,26,55]. For example, the R6/2 transgenic model that expresses the first exon of the human huntingtin gene containing ~150 CAG shows an aggressive HD-like phenotype that coincides with increased firing rate and decreased bursting activity [18]. Also, reduced correlated firing activity is observed when R6/2 mice freely explore an open-field arena [18]. Compared with R6/2 activity, HIP14 knockout neurons show similar changes in firing rate and proportion of correlated neuronal activity. However, given that the full-length HD transgenic model YAC128 and HIP14 show similar neurochemical changes in the striatum and motor behavioral deficits [15,39] evaluation of striatal neuronal activity in the full-length HD transgenic model YAC128 during plus-maze behavior is necessary to compare with the changes observed in HIP14 knockout mice. 

In conclusion, our results indicate that protein palmitoylation is a key component for proper striatal neuronal processing in vivo. Deficient palmitoylation by the HIP14 protein might underlie dysregulated neuronal processing involved with the motor and cognitive deficits that occur in neurodegenerative conditions such as HD. 
